# Optimality for set-valued optimization in the sense of vector and set criteria

**DOI:** 10.1186/s13660-017-1319-x

**Published:** 2017-02-17

**Authors:** Xiangyu Kong, GuoLin Yu, Wei Liu

**Affiliations:** Institute of Applied Mathematics, Beifang University of Nationalities, Yinchuan, Ningxia 750021 P.R. China

**Keywords:** 90C29, 90C46, 26B25, vector optimization, set optimization, optimality conditions, pseudoconvexity, Studniarski derivative

## Abstract

The vector criterion and set criterion are two defining approaches of solutions for the set-valued optimization problems. In this paper, the optimality conditions of both criteria of solutions are established for the set-valued optimization problems. By using Studniarski derivatives, the necessary and sufficient optimality conditions are derived in the sense of vector and set optimization.

## Introduction

Let *F* be a set-valued mapping from a set *S* to a real normed linear space *Y* and let $D\subset Y$ be a convex cone. The set-valued optimization problem is formalized as follows:

$$ \mbox{(P)} \quad \textstyle\begin{cases} \mbox{minimize}& F(x)\\ \mbox{subject to}& x\in S. \end{cases} $$

It is generally known that there are two types of criteria of solutions for problem (P): the vector criteria and set criteria. The vector criterion is the most commonly used in problem (P), which is looking for efficient elements of the set $F(S)=\bigcup_{x\in S} F(x)$. The problem (P) with vector criterion is often defined as set-valued vector optimization. Over the past decades, the set-valued vector optimization theory and its applications have been investigated extensively by many authors. Fruitful results are produced in this field, and we recommend the excellent books [1–4] and the references therein to the reader.

Another criterion, the set criterion, also called set optimization, was proposed by Kuroiwa [5, 6] in 1999 for the first time. Set optimization consists of looking for image sets $F(\bar{x})$, with $\bar{x}\in S$, which satisfy some set-relations between the rest of the image sets $F(x)$ with $x\in S$. In [3], p. 378, Jahn states that the set relation approach opens a new and exciting field of research. Although the set criterion seems to be more natural and interesting than the traditional one, the study of set optimization is very limited, such as papers on existence conditions, see [7–12]; on duality theory, see [13–16]; on optimality conditions, see [17–19]; on scalarization [20–23]; on well-posedness properties, see [24, 25]; on Ekeland variational principles, see [26, 27].

The investigation of optimality conditions, especially as regards the vector criterion, has received tremendous attention in the research of optimization problems and has been studied extensively. As everyone knows most of the problems under consideration are nonsmooth, which leads to introducing many kinds of generalized derivatives by the authors. A meaningful concept is the Studniarski derivative [28], which has some properties similar to the contingent derivative [1] and the Hadamard derivative [29]. Recently, much attention has been paid to optimality conditions and related topics for vector optimization by using Studniarski derivatives; see [30, 31].

Inspired by the above observations, in this paper, the optimality conditions are established for problem (P) in both the vector criterion and set criterion by using Studniarski derivatives. The rest of the paper is organized as follows: In Section 2, some well-known definitions and results used in sequel are recalled. In Section 3 and Section 4, the necessary and sufficient optimality conditions are given in the sense of the vector criterion and set criterion, respectively.

## Preliminaries

Throughout the rest of paper, it is assumed that *X* and *Y* are two real normed linear spaces, and $C\subset Y$ is a convex closed pointed cones with nonempty interior, *i.e.*, $\operatorname{int} C\neq\emptyset $. The partial order of *Y* is deduced by cone *C*. The ordering given by *C* on *Y* is denoted by ⩽, which is defined as follows:

$$\begin{aligned} y_{1}\leqslant y_{2} \quad \mbox{if } y_{2}-y_{1} \in C. \end{aligned}$$

Let $S\subset X$ be a nonempty set and $\bar{x}\in S$. The contingent cone to *S* at *x̄* is defined by (see [1]):

$$ T(S,\bar{x})=\bigl\{ x\in X: \exists(t_{n})\rightarrow0^{+}, (x_{n})\rightarrow x \mbox{ with } \bar{x}+t_{n} x_{n}\in S \mbox{ for all } n\in\mathbb{N}\bigr\} . $$

For a set-valued mapping $F: X\rightarrow2^{Y}$, the set

$$ \operatorname{dom}(F):=\bigl\{ x\in X: F(x)\neq\emptyset\bigr\} , $$

is called the domain of *F*, the set

$$ \operatorname{graph}(F):=\bigl\{ (x,y)\in X\times Y:\mbox{ }y\in F(x)\bigr\} $$

is called the graph of the map *F*, and the set

$$ \operatorname{epi}(F):=\bigl\{ (x,y)\in X\times Y:\mbox{ }y\in F(x)+C\bigr\} $$

is termed the epigraph of *F*. Let $(\bar{x},\bar{y})\in\operatorname{graph}(F)$, based upon the definition of contingent cone, we can derive $T(\operatorname{epi}(F),(\bar {x},\bar{y}))$, which is formulated by

2.1 $$\begin{aligned} T \bigl(\operatorname{epi}(F),(\bar{x},\bar{y}) \bigr):= {}& \bigl\{ (x,y)\in X\times Y: \exists(t_{n})\rightarrow0^{+}, (x_{n},y_{n}) \rightarrow (x,y) \\ &\mbox{with } \bar{y}+t_{n} y_{n}\in F( \bar{x}+t_{n} x_{n}) \mbox{ for all } n\in\mathbb{N} \bigr\} . \end{aligned}$$

### Definition 2.1

see [1]

Let $F: X\rightarrow2^{Y}$ be a set-valued map. *F* is called *C*-pseudoconvex at $(\bar{x},\bar{y})\in \operatorname{graph}(F)$ if

$$\operatorname{epi}(F)-(\bar{x},\bar{y})\subset T \bigl(\operatorname{epi}(F),(\bar{x},\bar {y}) \bigr). $$

For a set-valued mapping $F: X\rightarrow2^{Y}$, the upper and lower Studniarski derivatives of the map *F* is defined as follows:

### Definition 2.2

see [28]

Let $(\bar{x},\bar{y})\in\operatorname {graph}(F)$.

(i) The upper Studniarski derivatives of *F* at $(\bar{x},\bar {y})$ is defined by
  $$ \overline{D} F(\bar{x},\bar{y}) (x):=\limsup_{(t,x')\rightarrow (0^{+},x)} \frac{F(\bar{x}+tx')-\bar{y}}{t}. $$
(ii) The lower Studniarski derivative of *F* at $(\bar{x},\bar {y})$ is defined by
  $$ \underline{D} F(\bar{x},\bar{y}) (x):=\liminf_{(t,x')\rightarrow (0^{+},x)} \frac{F(\bar{x}+tx')-\bar{y}}{t}. $$

### Remark 2.3

Regarding the upper and lower Studniarski derivatives of the map *F*, one has:

- It is easily to see from Definition 2.2 that
  $$\begin{aligned} \overline{D} F(\bar{x},\bar{y}) (x)={}& \bigl\{ y\in Y: \exists (t_{n}) \rightarrow0^{+}, \exists(x_{n},y_{n})\rightarrow(x,y)\\ &\mbox{s.t. } \forall n, \bar{y}+t_{n} y_{n}\in F( \bar{x}+t_{n} x_{n}) \bigr\} , \\ \underline{D} F(\bar{x},\bar{y}) (x)={}& \bigl\{ y\in Y: \forall (t_{n}) \rightarrow0^{+}, \forall(x_{n})\rightarrow x, \exists (y_{n}) \rightarrow y\\ &\mbox{s.t. } \forall n, \bar{y}+t_{n} y_{n}\in F( \bar {x}+t_{n} x_{n}) \bigr\} . \end{aligned}$$
- The upper Studniarski derivative is an exactly contingent derivative [1] or an upper Hadamard derivative [29], and the lower Studniarski derivative is the lower Hadamard derivative introduced by Penot [29].

Lemma 2.4 is an useful property associated with *C*-pseudoconvexity of a set-valued mapping, which will be used in the next two sections.

### Lemma 2.4

*Let* $S\subset X$ *be a convex set and* $F: S\rightarrow2^{Y}$ *be* *C*-*pseudoconvex at* $(\bar{x},\bar{y})\in\operatorname {graph}(F)$. *One has*

2.2 $$ F(x)-\bar{y}\subset\overline{D}F(\bar{x},\bar{y}) (x-\bar{x})+C, \quad\textit{for all } x\in S. $$

### Proof

Since *F* at $(\bar{x},\bar{y})$ is *C*-pseudoconvex, it yields

$$ (x-\bar{x},y-\bar{y})\in T \bigl(\operatorname{epi}(F),(\bar{x},\bar{y}) \bigr),\quad \mbox{for all }x\in S, y\in F(x). $$

Therefore, we see from (2.1) that there exist $(t_{n})\rightarrow0^{+}$, $(x_{n},y_{n})\rightarrow(x-\bar{x},y-\bar{y})$ such that

$$ (\bar{x}+t_{n} x_{n},\bar{y}+t_{n} y_{n})\in\operatorname{epi}(F). $$

Thus

$$ \bar{y}+t_{n} y_{n}\in F(\bar{x}+t_{n} x_{n})+C. $$

Then one has

$$ y_{n}\in\frac{F(\bar{x}+t_{n} x_{n})-\bar{y}}{t_{n}}+C. $$

By taking the limits when $n\rightarrow+\infty$, we derive

$$ y-\bar{y}\in\overline{D}F(\bar{x},\bar{y}) (x-\bar{x})+C, \quad\mbox{for all } x\in S, y\in F(x). $$

So, we get (2.2), as desired. □

### Example 2.5

Let $X=Y=\mathbb{R}$, $C=\mathbb{R}_{+}$ and $F: X\rightarrow2^{Y}$ be defined by

$$ F(x):=\bigl\{ y\in Y: y\geq x^{2}\bigr\} , \quad\mbox{for all } x\in X. $$

Taking $(0,0)=(\bar{x},\bar{y})\in\operatorname{graph}(F)$, we can derive that

$$ \overline{D}F(0;0) (x)=\underline{D}F(0;0) (x)=\mathbb{R}_{+},\quad \mbox{for all } x \in X. $$

On the other hand, it is obviously that *F* is *C*-pseudoconvex at $(\bar{x},\bar{y})=(0,0)$ and satisfies

$$ F(x)-\bar{y}\subset\overline{D}F(\bar{x},\bar{y}) (x-\bar{x})+C, \quad\mbox{for all } x\in X. $$

## Optimality conditions with vector criterion

Let $S\subset X$ be a nonempty set, $F: S\rightarrow2^{Y}$ be a set-valued mapping and $(\bar{x},\bar{y})\in\operatorname{graph}(F)$.

*From now on, for convention we always assume that the upper and lower Studniarski derivatives of the map* *F* *at* $(\bar{x},\bar{y})$ *exist and* $\operatorname{dom}\overline{D}F(\bar{x},\bar{y})= \operatorname {dom}\underline{D}F(\bar{x},\bar{y})=S$.

Consider the set-valued optimization problem (P) formulated in Section 1. We shall establish the optimality conditions for vector criteria of solutions for problem (P). In order to distinguish from the case of set criteria, we rewrite the problem (P) as follows:

$$ \mbox{(VP)} \quad \textstyle\begin{cases} \min_{\leqslant}& F(x)\\ \mbox{s.t.}& x\in S\subset X. \end{cases} $$

### Definition 3.1

The pair $(\bar{x},\bar{y})\in\operatorname {graph}(F)$ is said to be a weak minimizer of (VP), denoted by $\bar{y}\in\operatorname {WMin}[F(S),C]$, if

$$ \bigl(F(S)-\bar{y} \bigr)\cap (-\operatorname{int}C )=\emptyset. $$

Theorems 3.2 and 3.3 are necessary optimality conditions for the weak minimizer of problem (VP).

### Theorem 3.2

*Let* $(\bar{x},\bar{y})\in\operatorname{graph}(F)$ *be a weak minimizer of* (VP). *Then*

3.1 $$ \bigl(\overline{D}F(\bar{x},\bar{y}) (x) \bigr)\cap (-\operatorname{int}C )=\emptyset,\quad \forall x\in S. $$

### Proof

Let $y\in\overline{D}F(\bar{x},\bar{y})(x)$ with $x\in S$. Then there exist $(t_{n})\rightarrow0^{+}$, $(x_{n})\rightarrow \bar{x}$ and $y_{n}\in F(\bar{x}+t_{n} x_{n})$ such that

3.2 $$ \frac{y_{n}-\bar{y}}{t_{n}}\rightarrow y. $$

Because $(\bar{x},\bar{y})$ is a weak minimizer of (VP), one has

3.3 $$ \bigl(F(\bar{x}+t_{n} x_{n})-\bar{y} \bigr)\cap (- \operatorname{int}C )=\emptyset. $$

Now, let us show that $y\notin-\operatorname{int}C$. In fact, assuming that $y\in-\operatorname{int}C$, since $-\operatorname{int}C$ is open, we obtain from (3.2)

$$ y_{n}-\bar{y}\in-\operatorname{int}C, \quad\mbox{for large }n. $$

Hence, it follows from $y_{n}\in F(\bar{x}+t_{n} x_{n})$ that

$$ y_{n}-\bar{y}\in \bigl(F(\bar{x}+t_{n} x_{n})- \bar{y} \bigr)\cap-\operatorname{int}C,\quad \mbox{for large }n, $$

which contradicts (3.3). □

### Theorem 3.3

*Let* $(\bar{x},\bar{y})\in\operatorname{graph}(F)$. *If* $(\bar{x},\bar{y})$ *is a weak minimizer of* (VP), *then*

3.4 $$ \bigl(\underline{D}F(\bar{x},\bar{y}) (x) \bigr)\cap (-\operatorname{int} C )=\emptyset,\quad \textit{for all } x\in T(S,\bar{x}). $$

### Proof

According to Theorem 3.2, one shows that for all $x\in S$

3.5 $$ \bigl(\overline{D}F(\bar{x},\bar{y}) (x) \bigr)\cap(-\operatorname{int} C)=\emptyset. $$

We proceed by contradiction. If the conclusion in Theorem 3.3 does not hold, then there exists $\hat{x}\in T(S,\bar{x})$ such that

3.6 $$ \underline{D}F(\bar{x},\bar{y}) (\hat{x})\cap(-\operatorname{int} C)\neq \emptyset. $$

Noticing that $\hat{x}\in T(S,\bar{x})$, there exist $(t_{n})\rightarrow 0^{+}$ and $(x_{n})\rightarrow x$ such that $\bar{x}+t_{n} x_{n}\in S$ for all *n*. In addition, we see from (3.6) that there exists $\hat{y}\in \underline{D}F(\bar{x},\bar{y})(\hat{x})\cap(-\operatorname{int} C)$. Hence, with the above $(t_{n})$ and $(x_{n})$ there is $(y_{n})\rightarrow\hat{y}$ such that

$$ \bar{y}+t_{n}y_{n}\in F(\bar{x}+t_{n} x_{n}). $$

So, we see that there exist $(t_{n})\rightarrow0^{+}$, $(x_{n})\rightarrow x$, and $(y_{n})\rightarrow\hat{y}$ such that

$$ \bar{y}+t_{n}y_{n}\in F(\bar{x}+t_{n} x_{n}). $$

Thus,

$$ \hat{y}\in \bigl(\overline{D}F(\bar{x},\bar{y}) (\hat{x}) \bigr)\cap (- \operatorname{int} C), $$

which is a contradiction to (3.5). □

Now, we present a sufficient optimality condition for problem (VP) under the assumption of pesudoconvexity of *F*.

### Theorem 3.4

*Let* *S* *be a convex set and* *F* *be* *C*-*pseudoconvex at* $(\bar{x},\bar{y})\in\operatorname{graph}(F)$. *If*

3.7 $$ \bigl(\overline{D}F(\bar{x},\bar{y}) (x-\bar{x}) \bigr)\cap (-\operatorname {int}C )= \emptyset, \quad\forall x\in S, $$

*then* $(\bar{x},\bar{y})$ *is a weak minimizer of* (VP).

### Proof

We proceed by contradiction. Suppose that $(\bar {x},\bar{y})$ is not a weak minimizer of (VP), then there exists $x\in S$ such that

3.8 $$ \bigl(F(x)-\bar{y} \bigr)\cap (-\operatorname{int}C )\neq\emptyset. $$

Since *F* is *C*-pseudoconvex at $(\bar{x},\bar{y})$, one shows from Lemma 2.4 that

$$ F(x)-\bar{y}\subset\overline{D}F(\bar{x},\bar{y}) (x-\bar {x})+C. $$

Therefore, one shows from (3.8) that

$$ \bigl(\overline{D}F(\bar{x},\bar{y}) (x-\bar{x})+D \bigr)\cap (-\operatorname {int}C ) \neq\emptyset, $$

and as a consequence

$$ \bigl(\overline{D}F(\bar{x},\bar{y}) (x-\bar{x}) \bigr)\cap (-\operatorname {int}C )\neq \emptyset. $$

This is a contradiction to (3.7). □

## Optimality conditions with set criterion

This section works with the optimality conditions for problem (P) in the sense of the set criterion. Firstly, let us recall the concepts of set relations.

### Definition 4.1

see [5]

Let *A* and *B* be two nonempty sets of *Y*. We write $A\leqslant^{\mathrm{l}} B$, if for all $b\in B$ there exists $a\in A$ such that $a\leqslant b$. Here, ‘$\leqslant^{\mathrm{l}}$’ is called a lower relation.

### Remark 4.2

The above defined lower relation is equivalent to $B\subset A+C$ and it is the generalization of the order induced by a pointed convex cone *C* in *Y*, in the following sense: $a\leqslant b$ if $b\in a+C$.

### Definition 4.3

see [14]

Let *A* and *B* be two nonempty sets of *Y*. We write $A< ^{\mathrm{lw}} B$, if $B\subset A+\operatorname {int}C$. Here, ‘$< ^{\mathrm{lw}}$’ is named a lower weak relation.

### Definition 4.4

see [14]

Let $\mathcal{A}$ be a family of subsets of *Y*.

1. $A\in\mathcal{A}$ is named a lower minimal of $\mathcal {A}$, if for each $B\in\mathcal{A}$ such that $B\leqslant^{\mathrm{l}} A$, it satisfies $A\leqslant^{\mathrm{l}} B$;
2. $A\in\mathcal{A}$ is called a lower weak minimal of $\mathcal{A}$, if for each $B\in\mathcal{A}$ such that $B< ^{\mathrm{lw}} A$, it satisfies $A< ^{\mathrm{lw}} B$.

The relation ‘$\leqslant^{\mathrm{l}}$’ determines in $\mathcal{A}$ the equivalence relation:

$$ A\sim B \quad\Leftrightarrow \quad A\leqslant^{\mathrm{l}} B\quad \mbox{and}\quad B \leqslant^{\mathrm{l}} A $$

whose classes it is denoted by $[A]^{\mathrm{l}}$. Analogously the relation ‘$< ^{\mathrm{lw}}$’ determines in $\mathcal{A}$ an equivalence relation whose classes are written $[A]^{\mathrm{lw}}$.

In the sense of a set criterion, the problem (P) can be rewritten in the next form:

$$ \mbox{(SP)}\quad \textstyle\begin{cases} \min_{\leqslant^{\mathrm{l}}}& \{F(x)\}\\ s.t.& x\in S\subset X. \end{cases} $$

In this case, a lower weak minimizer of the problem (SP) is a pair $(\bar{x}, F(\bar{x}) )$ such that $\bar{x}\in S$ and $F(\bar{x})$ is a lower weak minimal of the family of images of *F*, *i.e.* the family

$$ \mathcal{F}= \bigl\{ F(x): x\in S \bigr\} . $$

We say that $F(\bar{x})$ is a lower weak minimal of (SP) instead of $\mathcal{F}$.

### Example 4.5

Let $S=[0,1]\subset\mathbb{R}$, $Y=\mathbb {R}^{2}$ and $C=\mathbb{R}^{2}_{+}$. Let $F_{0}: [0,1]\rightarrow2^{\mathbb {R}^{2}}$ be a set-valued map and defined by

$$ F_{0}(x)= \textstyle\begin{cases} \{(y_{1},y_{2})\in\mathbb{R}^{2}: (y_{1}-1)^{2}+(y_{2}-1)^{2}=1\}, & x=0,\\ [(x,-x+1),(1,-x+1)],& 0< x< 1,\\ [(0,1),(1,0)],& x=1, \end{cases} $$

where $[y,y']$ with $y,y'\in\mathbb{R}^{2}$ denotes the line segment, *i.e.* $[y,y']=\{\lambda y+(1-\lambda)y': 0\leq\lambda\leq1\} $. Considering the following problem (SP)_0_:

$$ \mbox{(SP)}_{0} \quad \textstyle\begin{cases} \min_{\leqslant^{\mathrm{l}}}& \{F_{0}(x)\}\\ s.t.& x\in[0,1]. \end{cases} $$

By computing, we can derive that $(1, F(1))$ is the lower minimizer of (SP)_0_ and $(x,F(x))$ with $0< x \leq1$ is the lower weak minimizer of (SP)_0_.

### Definition 4.6

see [19]

Let $F(\bar{x})$ be a lower weak minimal of (SP). $F(\bar{x})$ is named strict lower weak minimal of (SP), if there exists a neighborhood $U(\bar{x})$ of *x̄* such that $F(x)\nless^{\mathrm{lw}} F(\bar{x})$, for all $x\in U(\bar{x})\cap S$. Then *x̄* is called a strict lower weak minimum of (SP).

### Lemma 4.7

see [19]

*Let* $\bar{x}\in S$. *Then* $F(\bar {x})$ *is a lower weak minimal of* (SP) *if and only if for each* $x\in S$ *one of the conditions below is fulfilled*:

1. $F(x)\in[F(\bar{x})]^{\mathrm{lw}}$.
2. *There exists* $\bar{y}\in F(\bar{x})$ *such that* $(F(x)-\bar{y} )\cap(-\operatorname{int}C)=\emptyset$.

### Definition 4.8

see [19]

(Domination property). It is called that a subset $A\subset Y$ has the *D*-weak minimal property, if for all $y\in A$ there exists a weakly minimal *a* of *A* such that $a-y\in(-\operatorname{int} C)\cup\{0\}$.

### Lemma 4.9

*Let* $\bar{x},x\in S$ *and* $\bar{y}\in F(\bar {x})$. *If* $F(x)\nless^{\mathrm{lw}} F(\bar{x})$, $\operatorname{WMin}[F(\bar{x}),C]=\{ \bar{y}\}$ *and* $F(\bar{x})$ *has the* *C*-*weak minimal property*, *one has*

4.1 $$ \bigl(F(x)-\bar{y} \bigr)\cap (-\operatorname{int}C )=\emptyset. $$

### Proof

Assuming that (4.1) does not hold, that is,

$$ \bigl(F(x)-\bar{y} \bigr)\cap (-\operatorname{int}C )\neq\emptyset. $$

Then there exist $y\in F(x)$ and $d\in\operatorname{int}C$ such that $y-\bar {y}=-d$. So, it yields $\bar{y}=y+d$ and $\bar{y}\in F(x)+\operatorname{int}C$. On the other hand, it follows from $\operatorname{WMin}[F(\bar{x}),C]=\{\bar {y}\}$ and the *C*-weak minimal property of $F(\bar{x})$ that, for all $\bar{y}'\in F(\bar{x})$, there is $d'\in\operatorname{int}C\cup\{0\}$ such that

$$ \bar{y}'=\bar{y}+d'\in F(x)+\operatorname{int}C. $$

Therefore, we derive that

$$ F(\bar{x})\subset F(x)+\operatorname{int}C, $$

which means $F(x)<^{\mathrm{lw}} F(\bar{x})$. This contradicts the hypothesis. □

### Theorem 4.10

*Let* *x̄* *be a strict lower weak minimum of* (SP). *If* $\operatorname{WMin}[F(\bar{x}),C]=\{\bar{y}\}$ *and* $F(\bar {x})$ *has the* *C*-*weak minimal property*, *then*

4.2 $$ \bigl(\overline{D}F(\bar{x},\bar{y}) (x) \bigr)\cap (-\operatorname{int}C )=\emptyset,\quad \textit{for all } x\in S. $$

### Proof

Because *x̄* is a strict lower weak minimum of (SP), we see from Definition 4.6 that there exists a neighborhood $U(\bar{x})$ of *x̄* such that $F(x')\nless^{\mathrm{lw}} F(\bar{x})$ for all $x'\in U(\bar{x})\cap S$. Let $x\in S$. Assuming that $y\in \overline{D}F(\bar{x},\bar{y})(x)$ then there exist $(t_{n})\rightarrow 0^{+}$, $(x_{n})\rightarrow\bar{x}$ and $y_{n}\in F(\bar{x}+t_{n} x_{n})$ such that

$$ \frac{y_{n}-\bar{y}}{t_{n}}\rightarrow y. $$

Therefore for large enough *n*, we can get $\bar{x}+t_{n} x_{n}\in U(\bar {x})\cap S$ which verifies $F(\bar{x}+t_{n} x_{n})\nless^{\mathrm{lw}} F(\bar{x})$. Thus, it follows from Lemma 4.9 that

4.3 $$ \bigl(F(\bar{x}+t_{n} x_{n})-\bar{y} \bigr)\cap(- \operatorname{int}C)=\emptyset, \quad\mbox{for large } n. $$

Let us prove that $y\notin-\operatorname{int}D$. Otherwise, one has

$$ y_{n}-\bar{y}\in-\operatorname{int}C,\quad \mbox{for large } n. $$

Noticing that $y_{n}\in F(\bar{x}+t_{n} x_{n})$, we derive

$$ y_{n}-\bar{y}\in \bigl(F(\bar{x}+t_{n} x_{n})- \bar{y} \bigr)\cap(-\operatorname {int}C),\quad \mbox{for large } n. $$

This is a contradiction to (4.3). So, we derive (4.2), as desired. □

### Theorem 4.11

*Let* *x̄* *be a strict lower weak minimum of* (SP). *If* $\operatorname{WMin}[F(\bar{x}),C]=\{\bar{y}\}$ *and* $F(\bar {x})$ *has the* *C*-*weak minimal property then*

4.4 $$ \underline{D}F(\bar{x},\bar{y}) (x)\cap(-\operatorname{int} C)=\emptyset,\quad \textit{for all } x\in T(S,\bar{x}). $$

### Proof

Based upon Theorem 4.10, we see that equation (4.2) holds. The rest of the proof can be followed by similar arguments to that of Theorem 3.3. □

### Theorem 4.12

*Let* *S* *be convex set and* *F* *be* *C*-*pseudoconvex at* $(\bar{x},\bar{y})\in\operatorname{graph}(F)$. *If for every* $x\in S$ *we have*

$$ \bigl(\overline{D}F(\bar{x},\bar{y}) (x-\bar{x}) \bigr)\cap (-\operatorname {int}C )= \emptyset, \quad\forall x\in S, $$

*then* *x̄* *is a lower weak minimum of* (SP).

### Proof

Suppose that *x̄* is not a lower weak minimum of (SP), it follows from Lemma 4.7 that there exists $x'\in S$ such that for each $\bar{y}'\in F(\bar{x})$ there is $y'\in F(x')$ with

4.5 $$ y'-\bar{y}'\in-\operatorname{int} C. $$

Since *F* is *C*-pseudoconvex at $(\bar{x},\bar{y})$, we derive from Lemma 2.4 that

$$ F\bigl(x'\bigr)-\bar{y}\subset\overline{D}F(\bar{x},\bar{y}) \bigl(x'-\bar{x}\bigr)+C. $$

Therefore, we obtain

4.6 $$ y'-\bar{y}\in\overline{D}F(\bar{x},\bar{y}) \bigl(x'- \bar{x}\bigr)+C. $$

Combining (4.5) with (4.6), we get

$$ \bigl(\overline{D}F(\bar{x},\bar{y}) \bigl(x'-\bar{x}\bigr)+D \bigr)\cap(-\operatorname {int}C)\neq\emptyset, $$

furthermore,

$$ \overline{D}F(\bar{x},\bar{y}) \bigl(x'-\bar{x}\bigr)\cap(- \operatorname{int}D)\neq \emptyset, $$

which is a contradiction. □

### Remark 4.13

By comparing the results derived in Section 3, it can be found that the optimality for the set criterion and vector criterion, with the suitable conditions, possesses the same forms in terms of Studniarshi derivatives.

## Conclusions

We have studied the optimality conditions for both the vector criterion and set criterion for a set-valued optimization problem in this note. We have presented two sufficient optimality conditions and a necessary condition for a weak minimizer in terms of Studniarski derivatives. In the set optimization criterion, utilizing the known results, we have proved two necessary optimality conditions for a strict lower weak minimum and a lower weak minimum. A sufficient optimality condition has been proved under the assumption of pseudoconvexity.
